# Prognostic impact of clinical factors for immune checkpoint inhibitor with or without chemotherapy in older patients with non-small cell lung cancer and PD-L1 TPS ≥ 50%

**DOI:** 10.3389/fimmu.2024.1348034

**Published:** 2024-02-23

**Authors:** Shota Takei, Hayato Kawachi, Tadaaki Yamada, Motohiro Tamiya, Yoshiki Negi, Yasuhiro Goto, Akira Nakao, Shinsuke Shiotsu, Keiko Tanimura, Takayuki Takeda, Asuka Okada, Taishi Harada, Koji Date, Yusuke Chihara, Isao Hasegawa, Nobuyo Tamiya, Yuki Katayama, Naoya Nishioka, Kenji Morimoto, Masahiro Iwasaku, Shinsaku Tokuda, Takashi Kijima, Koichi Takayama

**Affiliations:** ^1^ Department of Pulmonary Medicine, Graduate School of Medical Science, Kyoto Prefectural University of Medicine, Kyoto, Japan; ^2^ Department of Thoracic Oncology, Osaka International Cancer Institute, Osaka, Japan; ^3^ Department of Respiratory Medicine and Hematology, School of Medicine, Hyogo Medical University, Nishinomiya, Japan; ^4^ Department of Respiratory Medicine, Fujita Health University School of Medicine, Toyoake, Japan; ^5^ Department of Respiratory Medicine, Fukuoka University Hospital, Nanakuma, Japan; ^6^ Department of Respiratory Medicine, Japanese Red Cross Kyoto Daiichi Hospital, Kyoto, Japan; ^7^ Department of Respiratory Medicine, Japanese Red Cross Kyoto Daini Hospital, Kyoto, Japan; ^8^ Department of Respiratory Medicine, Saiseikai Suita Hospital, Suita, Japan; ^9^ Department of Medical Oncology, Fukuchiyama City Hospital, Fukuchiyama, Japan; ^10^ Department of Pulmonary Medicine, Kyoto Chubu Medical Center, Nantan, Japan; ^11^ Department of Respiratory Medicine, Uji-Tokushukai Medical Center, Uji, Japan; ^12^ Department of Respiratory Medicine, Saiseikai Shigaken Hospital, Rittou, Japan; ^13^ Department of Respiratory Medicine, Rakuwakai Otowa Hospital, Kyoto, Japan

**Keywords:** chemoimmunotherapy, immunochemotherapy, immune checkpoint inhibitor, non-small-cell lung cancer, PD-L1

## Abstract

**Introduction:**

The proportion of older patients diagnosed with advanced-stage non-small cell lung cancer (NSCLC) has been increasing. Immune checkpoint inhibitor (ICI) monotherapy (MONO) and combination therapy of ICI and chemotherapy (COMBO) are standard treatments for patients with NSCLC and programmed cell death ligand-1 (PD-L1) tumor proportion scores (TPS) ≥ 50%. However, evidence from the clinical trials specifically for older patients is limited. Thus, it is unclear which older patients benefit more from COMBO than MONO.

**Methods:**

We retrospectively analyzed 199 older NSCLC patients of Eastern Cooperative Oncology Group performance status (ECOG PS) 0-1 and PD-L1 TPS ≥ 50% who were treated with MONO or COMBO. We analyzed the association between treatment outcomes and baseline patient characteristics in each group, using propensity score matching.

**Results:**

Of the 199 patients, 131 received MONO, and 68 received COMBO. The median overall survival (OS; MONO: 25.2 vs. COMBO: 42.2 months, P = 0.116) and median progression-free survival (PFS; 10.9 vs. 11.8 months, P = 0.231) did not significantly differ between MONO and COMBO group. In the MONO group, OS was significantly shorter in patients without smoking history compared to those with smoking history [HR for smoking history against non-smoking history: 0.36 (95% CI: 0.16-0.78), P = 0.010]. In the COMBO group, OS was significantly shorter in patients with PS 1 than those with PS 0 [HR for PS 0 against PS 1: 3.84 (95% CI: 1.44-10.20), P = 0.007] and for patients with squamous cell carcinoma (SQ) compared to non-squamous cell carcinoma (non-SQ) [HR for SQ against non-SQ: 0.17 (95% CI: 0.06-0.44), P < 0.001]. For patients with ECOG PS 0 (OS: 26.1 months vs. not reached, P = 0.0031, PFS: 6.5 vs. 21.7 months, P = 0.0436) or non-SQ (OS: 23.8 months vs. not reached, P = 0.0038, PFS: 10.9 vs. 17.3 months, P = 0.0383), PFS and OS were significantly longer in the COMBO group.

**Conclusions:**

ECOG PS and histological type should be considered when choosing MONO or COMBO treatment in older patients with NSCLC and PD-L1 TPS ≥ 50%.

## 1 Introduction

Lung cancer is the leading cause of cancer-related deaths worldwide (1). Non-small-cell lung cancer (NSCLC) accounts for approximately 80% of all lung cancer cases, with the majority of NSCLC cases being diagnosed at an advanced, unresectable, and metastatic disease stage (2). Immune checkpoint inhibitors (ICIs), such as programmed death 1 (PD-1) or programmed death ligand 1 (PD-L1) antibodies, have improved the prognosis of advanced NSCLC. The PD-L1 tumor proportion score (TPS) is used as a predictor associated with treatment response (3, 4). In several phase 3 clinical trials, ICI monotherapy (MONO) has shown outstanding efficacy compared to chemotherapy in patients with NSCLC and PD-L1 TPS ≥ 50% and has been established as a standard first-line treatment option for such patients (5, 6). Concurrently, numerous phase 3 clinical trials for advanced NSCLC, regardless of PD-L1 TPS, have reported significantly improved clinical outcomes in patients treated with combination therapy of ICI and chemotherapy (COMBO) compared to those treated with chemotherapy alone. COMBO is thus considered as a standard first-line treatment for NSCLC (7–10). Similar to MONO, an association between PD-L1 TPS and therapeutic response has been reported in patients who received COMBO. Long-term follow-up analyses have shown favorable treatment responses particularly in the PD-L1 TPS ≥50% population (11, 12). Therefore, both MONO and COMBO are effective first-line treatment options for NSCLC patients with PD-L1 TPS ≥ 50%.

The proportion of older patients diagnosed with advanced-stage lung cancer has been increasing owing to the aging of the population (13), with almost half of all lung cancer cases occurring in patients aged 70 years or more (14). Consequently, formulating a treatment strategy for older patients with NSCLC is essential. However, evidence based on the clinical trials specifically for older patients is limited.

Pooled analyses of clinical trials involving pembrolizumab monotherapy in patients with PD-L1-positive advanced NSCLC reported a longer overall survival (OS) and superior safety profiles compared to standard chemotherapy in older patients with PD-L1 TPS ≥ 50%. However, approximately 40% of the older patients with NSCLC still experience mortality within 1 year, thus limiting the benefits of pembrolizumab monotherapy in this subgroup (15). Nonetheless, data on the efficacy and safety of COMBO for older patients are lacking, as COMBO may lead to more severe adverse events in older patients and is currently recommended only for a select group of patients (16). As such, the question of whether MONO or COMBO should be the preferred treatment choice for older patients with NSCLC and PD-L1 TPS ≥ 50% remains unanswered.

To this end, in this retrospective study, we conducted a real-world assessment to compare the efficacy and safety of MONO and COMBO in older patients with NSCLC and PD-L1 TPS ≥ 50%. In this study, we aimed to provide clarity on which clinical populations benefit from the addition of chemotherapy to ICI treatment.

## 2 Materials and methods

### 2.1 Patient population

This retrospective multicenter cohort study was conducted across 13 institutions in Japan. We collected the information on consecutive cases of advanced NSCLC (stage IV, including postoperative recurrence, according to the American Joint Committee on Cancer Staging Manual, version 8) in patients with PD-L1 TPS ≥ 50% who received first-line MONO or COMBO treatment between March 2017 and June 2021. We analyzed the treatment outcomes in older patients with PD-L1 TPS ≥ 50% and an Eastern Cooperative Oncology Group performance status (ECOG PS) of 0–1, since patients with NSCLC with poor ECOG are generally ineligible for several phase 3 clinical trials involving COMBO (7–10). In this study, ‘older patients’ were defined as those aged 70 years or older, following criteria established in previous clinical trials (17–20). Clinical data relevant to first-line treatment were obtained from electronic medical records. PD-L1 TPS in tumor cells was assessed using PD-L1 immunohistochemistry with the 22C3 pharmDx antibody (clone 22C3; Dako North America, Inc, Carpinteria, CA, USA), in accordance with the regulations of each facility.

This study was approved by the Ethics Review Board of the Kyoto Prefectural University of Medicine and was conducted with consent from the Ethics Review Board of each hospital (approval no. ERB-C-2113). Informed patient consent was not required owing to the retrospective nature of the study.

### 2.2 Assessments of efficacy and safety

Initially, we investigated the association between patient characteristics, including OS, in patients receiving either MONO or COMBO. Furthermore, we identified factors significantly associated with MONO or COMBO outcomes and compared the outcomes between patients administered with MONO and those administered with COMBO, with or without the identified factors. Treatment response was evaluated using the Response Evaluation Criteria in Solid Tumors version 1.1. Progression-free survival (PFS) was defined as the time from the start of first-line treatment until the occurrence of progressive disease or death from any cause. OS was defined as the time from the start of first-line treatment until death from any cause. The incidence of adverse events was assessed according to the Common Terminology Criteria for Adverse Events (CTCAE) version 5.0. Data were collected until February 28, 2023.

For comparing treatment outcomes between the MONO and COMBO groups, we adjusted for significant differences in the baseline characteristics of patients using propensity score matching (PSM) for the following variables: age, sex, smoking status, ECOG PS, histologic profile, PD-L1 TPS, and cancer stage. Nearest neighbor matching was performed at a ratio of 1:1 without replacement and a caliper of 0.2.

### 2.3 Statistical analysis

The relationship between age and other patient characteristics was examined using the Fisher’s exact test or the chi-square test. PFS and OS were calculated using the Kaplan–Meier method and were compared using the log-rank test. The hazard ratios (HRs) and their corresponding 95% confidence intervals (Cis) were determined using a Cox proportional hazard model in both univariate and multivariate analyses. All statistical analyses were performed using EZR version 1.61 (Saitama Medical Center, Jichi Medical University, Saitama, Japan), which is a graphical user interface for R (version 4.2.2; The R Foundation for Statistical Computing, Vienna, Austria). More precisely, it is a modified version of R designed to add statistical functions frequently used in biostatistics. Statistical significance was set at P < 0.05.

## 3 Results

### 3.1 Patient characteristics

A total of 446 patients with PD-L1 TPS ≥ 50% who received MONO or COMBO were screened for enrollment. Among them, 204 patients younger than 70 years were excluded. In addition, 43 patients with poor PS (PS 2–4) were excluded, as only patients with PS 0–1 were examined in clinical trials for treatment with COMBO. Finally, a total of 199 patients aged 70 years or more with PD-L1 TPS ≥ 50% were enrolled in this study, of which 131 received MONO while 68 received COMBO as the first-line treatment (**Supplementary Figure 1**). All patients in the MONO group were treated with pembrolizumab monotherapy. In the COMBO group, 56 patients were treated with a regimen that included pembrolizumab and 12 patients were treated with a regimen that included atezolizumab. 29 patients were treated with a regimen that included pemetrexed. Five patients with epidermal growth factor receptor mutations and two with anaplastic lymphoma kinase fusion were included. The median follow-up time was 21.9 months for both treatment groups.

The baseline characteristics of the patients are summarized in **Table 1**. Compared with the COMBO group, the MONO group had a significantly higher number of patients with a history of smoking (54/68 patients or 79.4% vs. 118/131 patients or 90.1%, P = 0.049), and there were significant differences in cancer stage between the two groups. After PSM weighting, each group contained 52 matched patients, with no significant differences in baseline characteristics observed between the two matched groups (**Supplementary Table 1**). The median follow-up time was 22.4 months for both groups.

**Table 1:** Patient characteristics and demographics at baseline (N = 199).

Patient characteristics	MONO (N = 131)	COMBO (N = 68)	P value
Age (years)			
Median (range)	76 (70–90)	73 (70–86)	
<75 years	51 (38.9)	49 (72.1)	0.638
≧75 years	80 (61.1)	19 (27.9)	
Sex			
Male	107 (81.7)	50 (73.5)	0.202
Female	24 (18.3)	18 (26.5)	
Smoking status			
Never	13 (9.9)	14 (20.6)	0.049
Current or former smoker	118 (90.1)	54 (79.4)	
ECOG PS			
0	50 (38.2)	25 (36.8)	0.878
1	81 (61.8)	43 (63.2)	
Histology			
Squamous cell carcinoma	41 (31.3)	20 (29.4)	0.904
Adenocarcinoma	77 (58.8)	40 (58.8)	
Other	13 (9.9)	8 (11.8)	
PD-L1 status			
50–89%	75 (57.3)	47 (69.1)	0.125
90–100%	56 (42.7)	21 (30.9)	
Stage			
IVA	44 (33.6)	24 (35.3)	0.027
IVB	51 (38.9)	36 (52.9)	
Recurrence	36 (27.5)	8 (11.8)	
Liver metastasis	15 (11.5)	9 (13.2)	0.819
Brain metastasis	16 (12.2)	12 (17.6)	0.293
Treatment regimen			
Pembrolizumab	131 (100)		
CBDCA/nab-PTX/Pembrolizumab		27 (39.7)	
CBDCA/PEM/Pembrolizumab		23 (33.8)	
CDDP/PEM/Pembrolizumab		6 (8.8)	
CBDCA/PTX/BEV/Atezolizumab		7 (10.3)	
CBDCA/nab-PTX/Atezolizumab		5 (7.4)	

ICI, immune checkpoint inhibitor; PSM, propensity score matching; ECOG PS, Eastern Cooperative Oncology Group Performance Status; PD-L1, programmed death ligand 1; TPS, tumor proportion score; CBDCA, carboplatin; CDDP, cisplatin; PEM, pemetrexed; nab-PTX, nanoparticle albumin-bound paclitaxel; PTX, paclitaxel; BEV, bevacizumab.

### 3.2 Treatment outcomes in all patients

**Figure 1:**
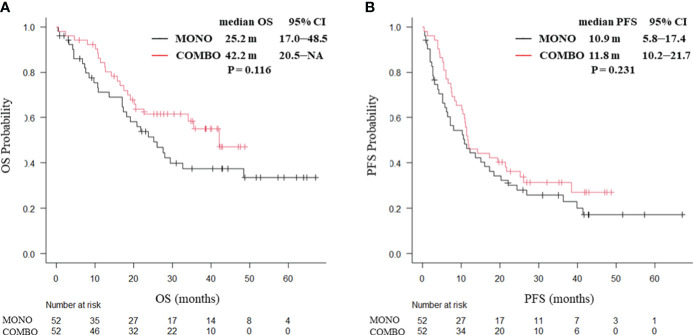
Kaplan–Meier curves for **(A)** OS and **(B)** PFS of patients aged ≥70 years with PD-L1 TPS ≥ 50% who received ICI monotherapy or combination therapy of ICI and chemotherapy after PSM (N = 104). OS, overall survival; PFS, progression-free survival; CI, confidence interval; PD-L1 TPS, programmed cell death ligand-1 tumor proportion score; ICI, immune checkpoint inhibitor; PSM, propensity score matching; MONO, ICI monotherapy; COMBO, ICI and chemotherapy combination therapy.

Overall, the median OS in the COMBO group appeared to be longer than that in the MONO group, though the difference was not statistically significant (42.2 vs. 25.0 months, P = 0.0502. Similarly, the median PFS (11.2 vs. 11.5 months, P = 0.683) did not significantly differ between the MONO and COMBO groups (**Supplementary Figure 2**). Adverse effects of CTCAE grade ≥ 3 were more frequently observed in the COMBO group (31/68, 45.6%) compared to the MONO group (39/131, 29.8%, P = 0.030). Analysis of the treatment outcomes after PSM showed that the median OS was similar between the MONO and COMBO groups (25.2 vs. 42.2 months, P = 0.116; **Figure 1A**). The median PFS was also similar between the two groups (10.9 vs. 11.8 months, P = 0.231; **Figure 1B**).

### 3.3 Treatment outcomes according to patient characteristics in each treatment group

We further analyzed the association of OS with patient characteristics within each treatment group. The results for the MONO group are presented in **Table 2**. Univariate analysis using Cox proportional hazards models indicated that none of the factors were significantly associated with OS. However, the multivariate Cox proportional hazards regression model revealed that smoking status (HR 0.36 [95% CI, 0.16–0.78], P = 0.010) was an independent predictor of OS.

**Table 2:** Cox proportional hazard models (multivariate analyses) for overall survival of patients aged ≥70 years with PD-L1 TPS ≥ 50% treated with ICI monotherapy (N = 131).

Characteristics	No of patients (%)	Overall survival (months)	Multivariate	
			HR (95% CI)	P-value
Age <75 years ≥75 years	51 (39) 80 (61)	27.7 23.8	1.02 (0.63–1.65) reference	0.923
Sex female male	24 (18) 107 (82)	Not reached 23.9	0.50 (0.23–1.06) reference	0.070
Smoking status Current or former smoker Never	118 (90) 13 (10)	27.7 16.5	0.36 (0.16–0.78) reference	0.010
ECOG PS 0 1	50 (38) 81 (62)	26.1 23.8	1.02 (0.63–1.66) reference	0.939
Histology Squamous cell carcinoma Non-squamous cell carcinoma	41 (31) 90 (69)	18.3 27.7	0.76 (0.46–1.25) reference	0.273
PD-L1 status 50–89% 90–100%	75 (57) 56 (43)	20.9 25.2	1.00 (0.62–1.62) reference	0.939
Stage IVA or IVB Recurrence	95 (73) 36 (27)	23.9 29.8	0.67 (0.39–1.17) reference	0.164

OS, overall survival; ICI, immune checkpoint inhibitor; ECOG PS, Eastern Cooperative Oncology Group Performance Status; PD-L1, programmed death ligand 1; TPS, tumor proportion score; HR, hazard risk; CI, confidence interval.

OS according to patient characteristics in the COMBO group is presented in **Table 3**. Univariate analysis using Cox proportional hazards models indicated that ECOG PS (HR 2.84 [95% CI, 1.16–6.98], P = 0.018) and histology (HR 0.32 [95% CI, 0.15–0.66], P = 0.0013) were significantly associated with OS. Similarly, the multivariate Cox proportional hazard regression model showed that ECOG PS (HR 3.84 [95% CI, 1.44¬10.20], P = 0.007) and histology (HR 0.17 [95% CI, 0.06–0.44, P < 0.001) were independent predictors of OS.

**Table 3:** Cox proportional hazard models (multivariate analyses) for overall survival in patients ≥70 years old with PD-L1 TPS ≥ 50% treated with combination therapy of ICI and chemotherapy (N = 68).

Characteristics	No of patients (%)	Overall survival (months)	Multivariate	
			HR (95% CI)	P-value
Age <75 years ≥75 years	49 (71) 19 (29)	Not reached 34.2	2.20 (0.95–5.09) reference	0.066
Sex female male	18 (25) 50 (75)	23.1 42.2	2.75 (0.75–10.03) reference	0.126
Smoking status Current or former smoker Never	54 (81) 14 (19)	42.2 23.1	1.07 (0.24–4.70) reference	0.929
ECOG PS 0 1	25 (37) 43 (63)	Not reached 34.2	3.84 (1.44–10.20) reference	0.007
Histology Squamous cell carcinoma Non-squamous cell carcinoma	20 (29) 48 (71)	18.5 Not reached	0.17 (0.06–0.44) reference	<0.001
PD-L1 status 50%–89% ≥90%	47 (68) 21 (32)	42.2 Not reached	0.47 (0.19–1.13) reference	0.091
Stage IVA or IVB Recurrence	60 (88) 8 (12)	42.2 Not reached	0.35 (0.09–1.38) reference	0.132

OS, overall survival; ICI, immune checkpoint inhibitor; ECOG PS, Eastern Cooperative Oncology Group Performance Status; PD-L1, programmed death ligand 1; TPS, tumor proportion score; HR, hazard risk; CI, confidence interval.

### 3.4 Treatment outcomes in patients with or without each independent predictor

We then compared the treatment outcomes of MONO and COMBO in patients with or without each identified independent clinical factor (smoking status, ECOG PS, and histology).

#### 3.4.1 Treatment outcomes in patients with or without smoking history

**Figure 2:**
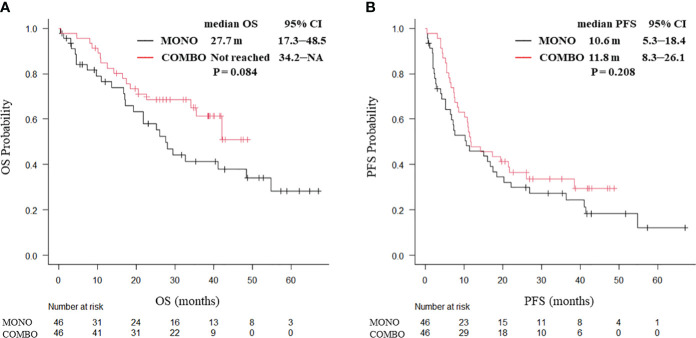
Kaplan–Meier curves for **(A)** OS and **(B)** PFS of patients aged ≥70 years with PD-L1 TPS ≥ 50% and with a smoking history, who received ICI monotherapy or combination therapy of ICI and chemotherapy after PSM (N = 92). OS, overall survival; PFS, progression-free survival; CI, confidence interval; PD-L1 TPS, programmed cell death ligand-1 tumor proportion score; ICI, immune checkpoint inhibitor; PSM, propensity score matching; MONO, ICI monotherapy; COMBO, ICI and chemotherapy combination therapy.

After PSM weighting, 92 patients with smoking history and 12 without smoking history were included. No significant differences in baseline characteristics of the patients were found between the two groups. In patients with a smoking history, no significant differences in the median OS (27.7 months vs. not reached, P = 0.084; **Figure 2A**) and median PFS (10.6 vs. 11.8 months, P = 0.208; **Figure 2B**) were observed between the MONO and COMBO groups. Similarly, in patients without a smoking history, no significant differences in the median OS (15.6 vs. 20.1 months, P = 0.252) and the median PFS (10.9 vs. 7.4 months, P = 0.43; **Supplementary Figure 3**) were observed.

#### 3.4.2 Treatment outcomes in patients with ECOG PS 0 or 1

**Figure 3:**
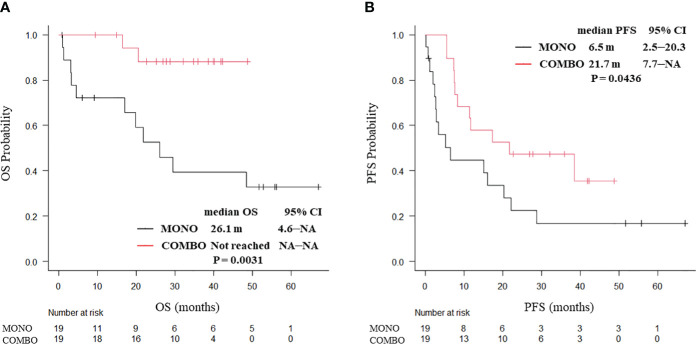
Kaplan–Meier curves for **(A)** OS and **(B)** PFS of ECOG PS 0 patients aged ≥70 years with PD-L1 TPS ≥ 50% who received ICI monotherapy or combination therapy of ICI and chemotherapy after PSM (N = 38). OS, overall survival; PFS, progression-free survival; CI, confidence interval; PD-L1 TPS, programmed cell death ligand-1 tumor proportion score; ICI, immune checkpoint inhibitor; PSM, propensity score matching; MONO, ICI monotherapy; COMBO, ICI and chemotherapy combination therapy.

PSM weighting, 38 patients with ECOG PS 0 and 62 patients with ECOG PS 1 were included. In ECOG PS 0 patients, both the median OS (not reached vs. 26.1 months, P = 0.0031; **Figure 3A**) and median PFS (21.7 vs. 6.5 months, P = 0.0436; **Figure 3B**) were significantly longer in the COMBO group than in the MONO group. By contrast, in ECOG PS1 patients, the median OS (25.5 vs. 35.5 months, P = 0.544) and the median PFS (11.5 vs. 12.0 months, P = 0.406; **Supplementary Figure 4**) were similar between the two groups.

#### 3.4.3 Treatment outcomes in patients with squamous cell carcinoma or non-squamous cell carcinoma

**Figure 4:**
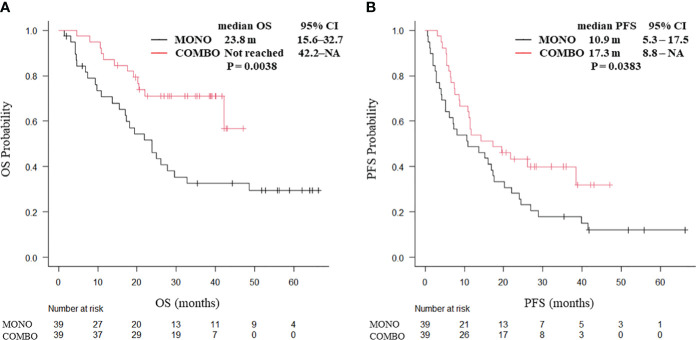
Kaplan–Meier curves for **(A)** OS and **(B)** PFS of patients with non-squamous cell carcinoma aged ≥70 years with PD-L1 TPS ≥ 50% who received ICI monotherapy or combination therapy of ICI and chemotherapy after PSM (N = 78). OS, overall survival; PFS, progression-free survival; CI, confidence interval; PD-L1 TPS, programmed cell death ligand-1 tumor proportion score; ICI, immune checkpoint inhibitor; PSM, propensity score matching; MONO, ICI monotherapy; COMBO, ICI and chemotherapy combination therapy.

After PSM weighting, 20 patients with squamous cell carcinoma and 78 with non-squamous cell carcinoma were included. In patients with squamous cell carcinoma, no significant differences were observed in the median OS (25.2 vs. 16.5 months, P = 0.566) and median PFS (9.0 vs. 10.2 months, P = 0.993; **Supplementary Figure 5**) between the MONO and COMBO groups. By contrast, both the median OS (not reached vs. 23.8 months, P = 0.0038; **Figure 4A**) and median PFS (17.3 vs. 10.9 months, P = 0.0383; **Figure 4B**) were significantly longer in the COMBO group than in the MONO group in patients with non-squamous cell carcinoma.

## 4 Discussion

Aging is a complex process characterized by the accrual of genetic and environmental factors, leading to a diverse array of alterations, such as diminished telomeres, genomic instability, impaired mitochondrial function, disruption of protein homeostasis, epigenetic modifications, declining immune system functionality, and the onset of cellular senescence. Furthermore, increasing age heightens the susceptibility to developing lung cancer, a phenomenon that is exacerbated by the growing aging population worldwide (21). Aging has also been reported to reduce the generation of new T cells from the thymus and impair T cell diversity, indicating that the adverse consequences of aging on T cells could potentially influence therapeutic efficacy (22).

In this study, we retrospectively analyzed the treatment outcomes in older patients with NSCLC and with PD-L1 TPS ≥ 50% who received MONO or COMBO as first-line treatment, both of which are recognized as standard first-line treatment options for patients with NSCLC and high PD-L1 TPS. Our findings indicated that there were no significant differences in PFS and OS between MONO and COMBO in patients with NSCLC aged ≥ 70 years with PD-L1 TPS ≥ 50%. However, notably, in patients with an ECOG PS 0 or those with non-squamous cell carcinoma, both PFS and OS were significantly longer in the COMBO group compared to the MONO group. To the best of our knowledge, this study is the first to identify clinical factors in an older NSCLC population that may benefit from COMBO rather than from MONO, which may be applied as predictors for clinical decision-making in this population.

Despite the valuable insights provided by our study, several limitations should be acknowledged. First, this study was a retrospective, nonrandomized study. Thus, the possibility of selection bias cannot be ruled out. However, we conducted PSM to reduce selection bias. Second, adverse events and complications are always a concern in chemotherapy for older patients, but this study did not mention either in detail. Third, in the MONO group, subsequent therapy after progression of first line pembrolizumab monotherapy may have affected the prognosis. However, our study had a lack of data about the detail of second line treatment, and we could not investigate these association. Finally, the study included only Japanese patients, and patient sample size was limited, essentializing the need for a global larger cohort to validate our novel findings.

In this study, the treatment efficacy did not differ between MONO and COMBO in the older population. A pooled analysis of phase 3 clinical trials previously reported that most patient subgroups with NSCLC and PD-L1 TPS ≥ 50% may achieve comparable or superior OS and PFS outcomes compared to MONO, whereas the outcomes of the subgroup analysis based on age in this study indicate that elderly patients receiving COMBO may not have improved outcomes over MONO (23). Additionally, recent analysis and real-world data reported MONO is generally preferred in older patients with NSCLC and PD-L1 TPS ≥ 50% (24, 25). In our subgroup analysis of patients ≥70 years, COMBO also did not show an improvement over MONO in OS or PFS outcomes, suggesting that the benefits of adding chemotherapy to MONO might be more limited in older patients with NSCLC compared to that in their younger counterparts. Based on these results, when considering the addition of chemotherapy to MONO for older NSCLC patients with high PD-L1 TPS, it becomes critical to appropriately select patients with predictive factors for better efficacy, particularly in this older demographic. Further studies are needed to identify clinical biomarkers that correlate with the treatment outcome for the efficacy and tolerability of COMBO in older patients.

Our study showed that COMBO yielded better clinical outcomes than MONO in patients with ECOG PS 0, while no significant difference was observed in patients with ECOG PS 1, suggesting that a more detailed assessment of general conditions at pretreatment may be useful for treatment selection in older patients. Similarly, the JCOG1210/WJOG7813L trial, in which carboplatin/pemetrexed treatment was compared with docetaxel treatment in older patients with NSCLC, indicated that the OS benefit for carboplatin/pemetrexed was notable in patients with ECOG PS 0 at baseline but not in those with ECOG PS 1 (26).

Given the results of this study, it may be necessary to subdivide PS and patient background when considering treatment strategies for older patients. ECOG PS is a simple and useful scoring system in a clinical setting. However, it is subject to high interobserver variability, and clinicians are more likely to evaluate better ECOG PS than patients themselves. ECOG PS can only assess patients from a functional perspective and may miss important impairments identified by the Geriatric Assessment (GA) (27). In recent years, several guidelines have recommended GA over ECOG PS for older patients, as it provides a comprehensive evaluation including physical, social, and spiritual aspects (28–30). Screening tools such as the G8, the Flemish version of the Triage Risk Screening Tool, and the Clinical Frailty Scale allow for a more detailed assessment of daily living and frailties of older patients (31, 32). In the field of geriatric oncology, several studies have reported the usefulness of these screening tools in prognosis prediction (33–35). Therefore, utilizing these tools may strongly assist in selecting better treatment choices for older patients with cancer.

The present study indicates that COMBO may be more effective than MONO in patients with non-squamous cell carcinoma. The tumor immune microenvironment, including the composition of CD8+ lymphocytes infiltrating the tumor, differs between squamous cell lung cancer and non-squamous cell carcinoma (36). In addition, the poor treatment efficacy of concomitant chemotherapy to MONO in older patients with squamous cell carcinoma is presumably based on a heavier smoking history and higher prevalence of comorbidities in these patients (37). Squamous cell carcinoma is strongly associated with aging and smoking; thus, patients with this carcinoma type are often prone to comorbidities, the most frequent of which are cardiovascular diseases and chronic obstructive pulmonary diseases (38). These comorbidities not only affect treatment choice and adherence to treatment but are also associated with poor survival outcome as comorbidity-related symptoms affect OS (39). Patients with severe comorbidities are more likely to experience hematologic toxicity after chemotherapy (40). For these reasons, the potential prognostic impact of comorbidities in squamous cell carcinoma of the lung is multifactorial, limiting the benefit of concomitant chemotherapy.

## 5 Conclusions

In conclusion, our study revealed that both PFS and OS were similar between MONO and COMBO treatments for patients with NSCLC aged ≥ 70 years with high PD-L1 TPS ≥ 50% but were significantly longer for COMBO than MONO in patients with ECOG PS 0 or non-squamous cell carcinoma. Therefore, ECOG PS and histological type may be important factors to consider when choosing between MONO or COMBO treatment in this population.

## Data availability statement

The original contributions presented in the study are included in the article/**Supplementary Material**. Further inquiries can be directed to the corresponding author.

## Ethics statement

The studies involving humans were approved by Ethics Review Board of the Kyoto Prefectural University of Medicine and the Ethics Review Board of each hospital (approval no. ERB-C-2113). The studies were conducted in accordance with the local legislation and institutional requirements. Written informed consent for participation was not required from the participants or the participants’ legal guardians/next of kin because owing to the retrospective nature of the study.

## Author contributions

ST: Conceptualization, Data curation, Formal analysis, Investigation, Methodology, Writing – original draft, Writing – review & editing. HK: Conceptualization, Data curation, Formal analysis, Investigation, Methodology, Writing – original draft, Writing – review & editing. TY: Conceptualization, Data curation, Formal analysis, Investigation, Methodology, Writing – original draft. Writing – review & editing. MT: Data curation, Writing – review & editing. YN: Data curation, Writing – review & editing. YG: Data curation, Writing – review & editing. AN: Data curation, Writing – review & editing. SS: Data curation, Writing – review & editing. KT: Data curation, Writing – review & editing. TT: Data curation, Writing – review & editing. AO: Data curation, Writing – review & editing. TH: Data curation, Writing – review & editing. KD: Data curation, Writing – review & editing. YC: Data curation, Writing – review & editing. IH: Data curation, Writing – review & editing. NT: Data curation, Writing – review & editing. YK: Writing – review & editing. NN: Writing – review & editing. KM: Writing – review & editing. MI: Writing – review & editing. ST: Writing – review & editing. TK: Writing – review & editing. KT: Supervision, Writing – review & editing.
