# Supplementary material for: Prognostic impact of clinical factors for immune checkpoint inhibitor with or without chemotherapy in older patients with non-small cell lung cancer and PD-L1 TPS ≥ 50%
Source: Front Immunol. 2024 Feb 23;15:1348034. doi: 10.3389/fimmu.2024.1348034 (PMC10920331; doi:10.3389/fimmu.2024.1348034)
Supplement: Supplementary file 1 [file Presentation_1.pptx]

## Slide 1
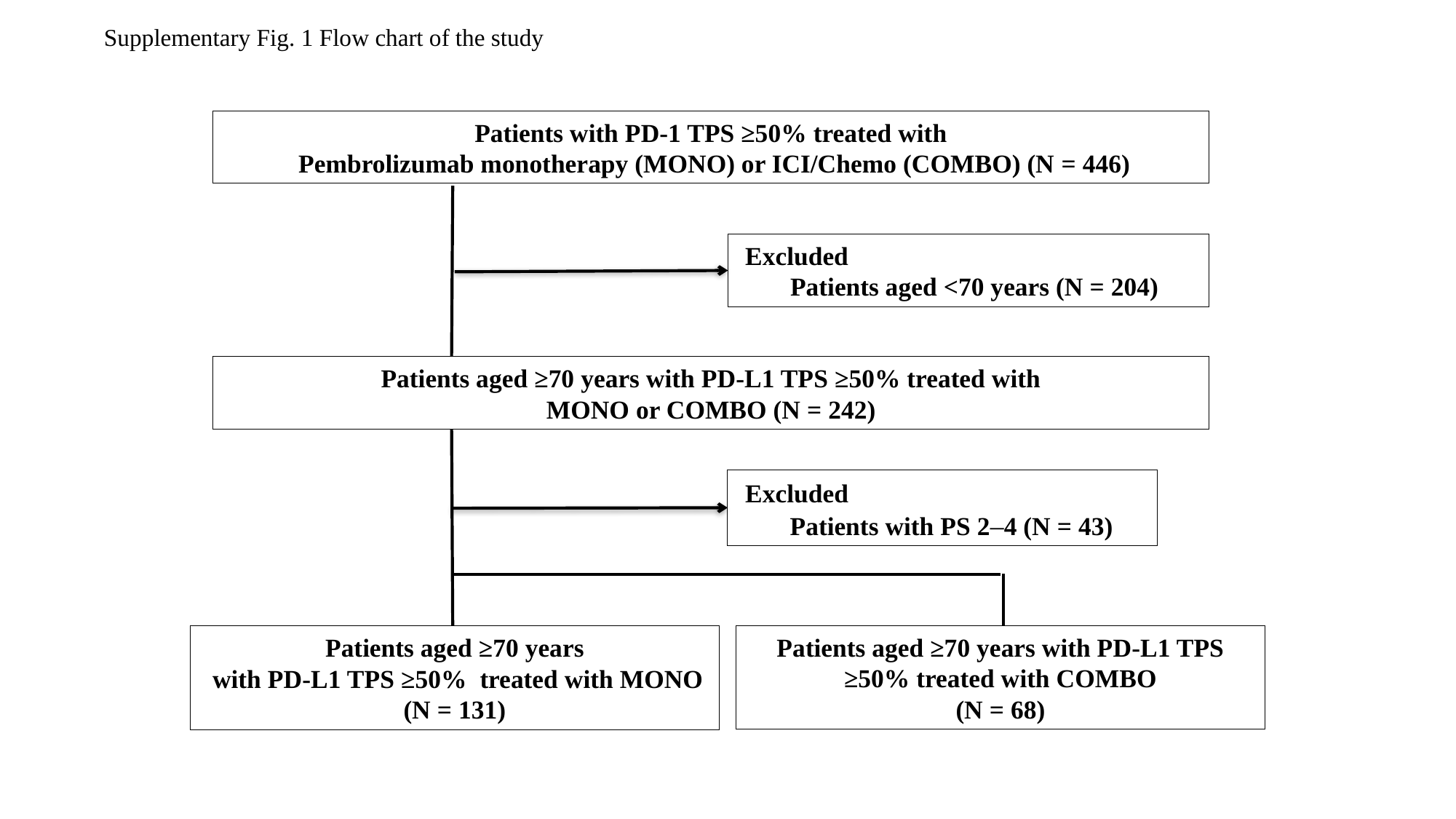

Supplementary Fig. 1 Flow chart of the study
Patients with PD-1 TPS ≥50% treated with
 Pembrolizumab monotherapy (MONO) or ICI/Chemo (COMBO) (N = 446)
 Excluded
 　Patients aged <70 years (N = 204)
Patients aged ≥70 years with PD-L1 TPS ≥50% treated with
MONO or COMBO (N = 242)
 Excluded
 　Patients with PS 2–4 (N = 43)
Patients aged ≥70 years with PD-L1 TPS ≥50% treated with COMBO
(N = 68)
Patients aged ≥70 years
 with PD-L1 TPS ≥50% treated with MONO
(N = 131)

## Slide 2
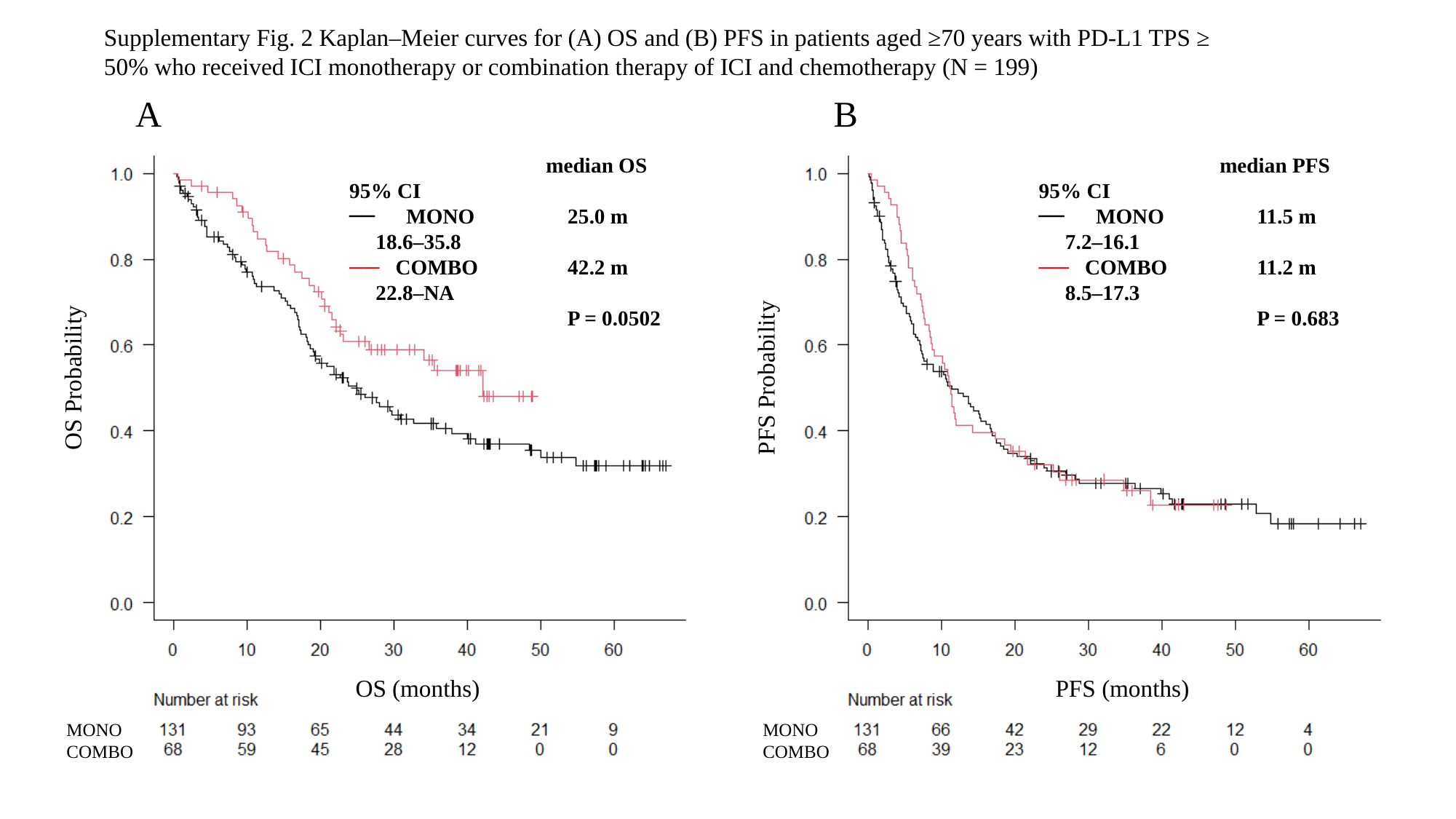

Supplementary Fig. 2 Kaplan–Meier curves for (A) OS and (B) PFS in patients aged ≥70 years with PD-L1 TPS ≥ 50% who received ICI monotherapy or combination therapy of ICI and chemotherapy (N = 199)
A
B
　　　　　　　　　median OS 　 95% CI
──　MONO	25.0 m　　　18.6–35.8
── COMBO	42.2 m　　　22.8–NA
　　　　　　　　　　P = 0.0502
　　　　　　　　 median PFS 　95% CI
──　MONO	11.5 m　　　7.2–16.1
── COMBO	11.2 m　　　8.5–17.3
　　　　　　　　　　P = 0.683
　　　　　　　　　　　median OS
----- MONO 　25.0 (months)
----- COMBO 　42.2 (
　　　　　　　　　　　median PFS
----- MONO 　11.5 (months)
----- COMBO 　11.2
OS Probability
PFS Probability
OS (months)
PFS (months)
MONO
COMBO
MONO
COMBO
MONO
COMBO
MONO
COMBO

## Slide 3
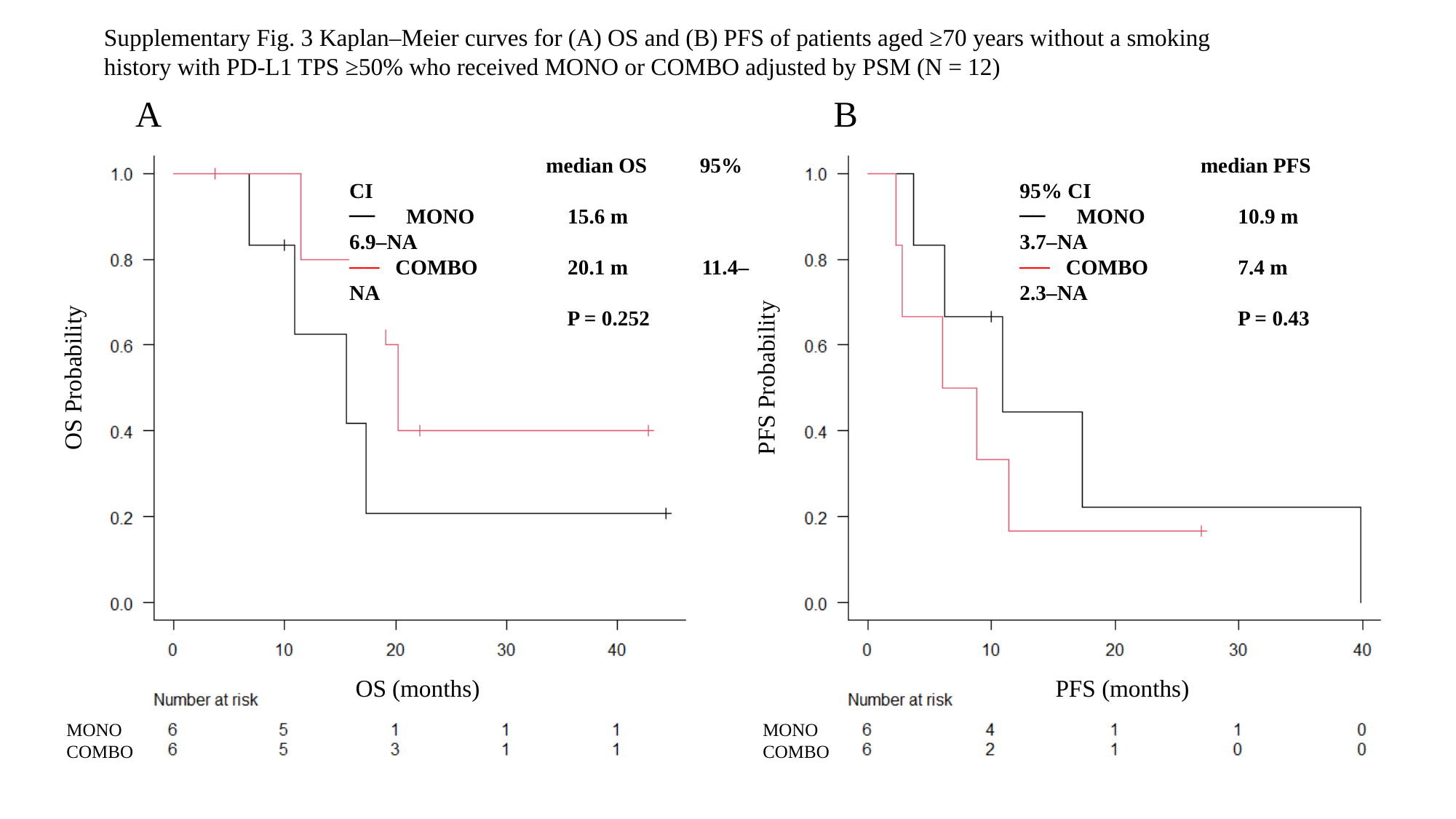

Supplementary Fig. 3 Kaplan–Meier curves for (A) OS and (B) PFS of patients aged ≥70 years without a smoking history with PD-L1 TPS ≥50% who received MONO or COMBO adjusted by PSM (N = 12)
A
B
　　　　　　　　　median OS 　 95% CI
──　MONO	15.6 m　　　 6.9–NA
── COMBO	20.1 m　　　11.4–NA
　　　　　　　　　　P = 0.252
　　　　　　　　 median PFS 　95% CI
──　MONO	10.9 m　　　3.7–NA
── COMBO	7.4 m　　　 2.3–NA
　　　　　　　　　　P = 0.43
OS Probability
PFS Probability
OS (months)
PFS (months)
MONO
COMBO
MONO
COMBO

## Slide 4
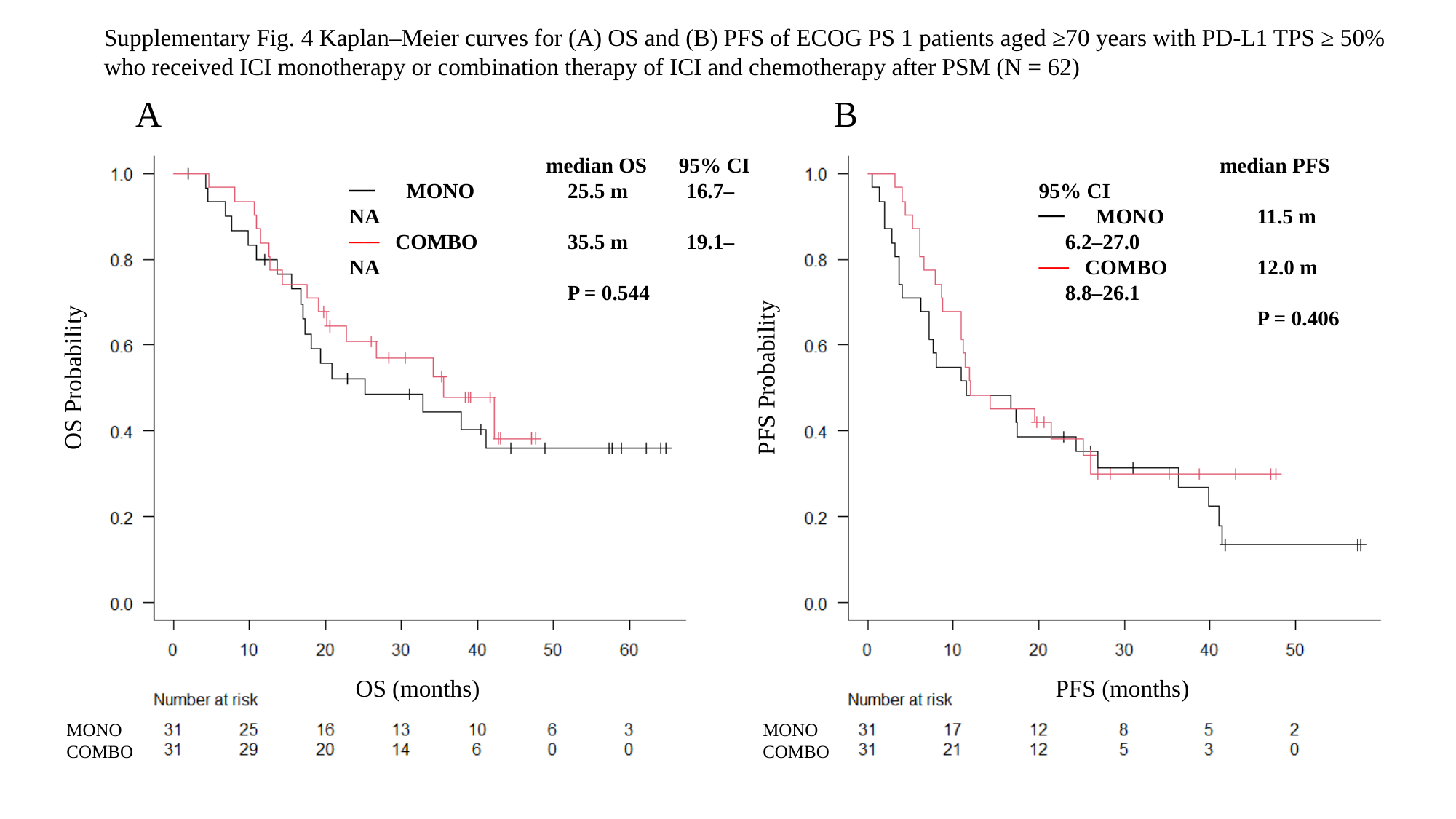

Supplementary Fig. 4 Kaplan–Meier curves for (A) OS and (B) PFS of ECOG PS 1 patients aged ≥70 years with PD-L1 TPS ≥ 50% who received ICI monotherapy or combination therapy of ICI and chemotherapy after PSM (N = 62)
A
B
　　　　　　　　　median OS 95% CI
──　MONO	25.5 m　　 16.7–NA
── COMBO	35.5 m　　 19.1–NA
　　　　　　　　　　P = 0.544
　　　　　　　　 median PFS 　95% CI
──　MONO	11.5 m　　　6.2–27.0
── COMBO	12.0 m　　　8.8–26.1
　　　　　　　　　　P = 0.406
OS Probability
PFS Probability
OS (months)
PFS (months)
MONO
COMBO
MONO
COMBO

## Slide 5
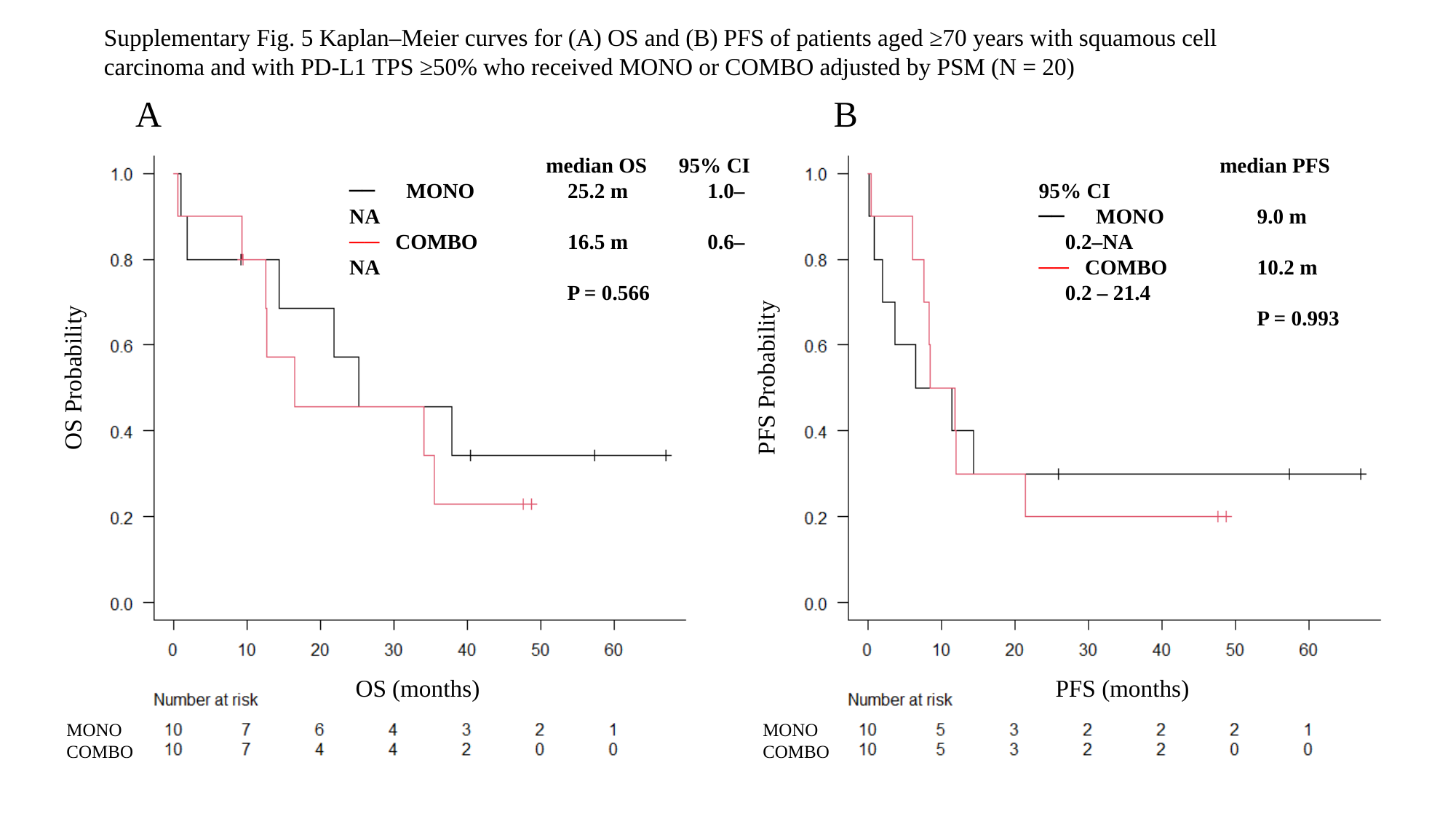

Supplementary Fig. 5 Kaplan–Meier curves for (A) OS and (B) PFS of patients aged ≥70 years with squamous cell carcinoma and with PD-L1 TPS ≥50% who received MONO or COMBO adjusted by PSM (N = 20)
A
B
　　　　　　　　　median OS 95% CI
──　MONO	25.2 m　　 　1.0–NA
── COMBO	16.5 m　　 　0.6–NA
　　　　　　　　　　P = 0.566
　　　　　　　　 median PFS 　95% CI
──　MONO	9.0 m　 　　0.2–NA
── COMBO	10.2 m　　　0.2 – 21.4
　　　　　　　　　　P = 0.993
OS Probability
PFS Probability
OS (months)
PFS (months)
MONO
COMBO
MONO
COMBO
