# Supplementary material for: Prognostic impact of clinical factors for immune checkpoint inhibitor with or without chemotherapy in older patients with non-small cell lung cancer and PD-L1 TPS ≥ 50%
Source: Front Immunol. 2024 Feb 23;15:1348034. doi: 10.3389/fimmu.2024.1348034 (PMC10920331; doi:10.3389/fimmu.2024.1348034)
Supplement: Supplementary file 2 [file Table_1.docx]

**Supplementary table.** Patient characteristics and demographics at baseline adjusted using PSM (N = 104).

| **Patient characteristics** | **MONO group**  **(N = 52)** | **COMBO group**  **(N = 52)** | **P value** |
| --- | --- | --- | --- |
| Age (years) |  |  |  |
| Median (range) | 74 (70–82) | 73 (70–86) |  |
| <75 years | 31 (59.6) | 33 (63–5) | 0.840 |
| ≥75 years | 21 (40.4) | 19 (36.5) |  |
| Sex |  |  |  |
| Male | 43 (82.7) | 44 (84.6) | 1.000 |
| Female | 9 (17.3) | 8 (15.4) |  |
| Smoking status |  |  |  |
| Never | 6 (11.5) | 5 (9.6) | 1.000 |
| Current or former | 46 (88.5) | 47 (90.4) |  |
| ECOG PS |  |  |  |
| 0 | 21 (40.4) | 21 (40.4) | 1.000 |
| 1 | 31 (59.6) | 31 (59.6) |  |
| Histology |  |  |  |
| Squamous cell carcinoma | 12 (23.1) | 17 (32.7) | 0.597 |
| Adenocarcinoma | 34 (65.4) | 29 (55.8) |  |
| Other | 6 (11.5) | 6 (11.5) |  |
| PD-L1 status |  |  |  |
| 50%–89% | 34 (65.4) | 32 (61.5) | 0.839 |
| 90%–100% | 18 (34.6) | 20 (38.5) |  |
| Stage |  |  |  |
| IVA | 17 (32.7) | 20 (38.5) | 0.798 |
| IVB | 28 (53.9) | 24 (46.2) |  |
| Recurrence | 7 (13.5) | 8 (15.4) |  |
| Liver metastasis | 4 (7.7) | 8 (15.4) | 0.358 |
| Brain metastasis | 10 (19.2) | 8 (15.4) | 0.796 |
| Treatment regimen |  |  |  |
| Pembrolizumab | 52 (100) |  |  |
| CBDCA/nab-PTX/Pembrolizumab |  | 21 (40.4) |  |
| CBDCA/PEM/Pembrolizumab |  | 17 (32.7) |  |
| CDDP/PEM/Pembrolizumab |  | 3 (5.8) |  |
| CBDCA/PTX/BEV/Atezolizumab |  | 6 (11.5) |  |
| CBDCA/nab-PTX/ Atezolizumab |  | 5 (9.6) |  |

PSM, propensity score matching; ECOG PS, Eastern Cooperative Oncology Group Performance Status; PD-L1, programmed death ligand 1; TPS, tumor proportion score; CBDCA, carboplatin; CDDP, cisplatin; PEM, pemetrexed; nab-PTX, nanoparticle albumin-bound paclitaxel; PTX, paclitaxel; BEV, bevacizumab.
